# Depression and Accelerated Aging: The Eveningness Chronotype and Low Adherence to the Mediterranean Diet Are Associated with Depressive Symptoms in Older Subjects

**DOI:** 10.3390/nu17010104

**Published:** 2024-12-30

**Authors:** José V. Sorlí, Edurne de la Cámara, Rebeca Fernández-Carrión, Eva M. Asensio, Olga Portolés, Carolina Ortega-Azorín, Alejandro Pérez-Fidalgo, Laura V. Villamil, Montserrat Fitó, Rocío Barragán, Oscar Coltell, Dolores Corella

**Affiliations:** 1 Department of Preventive Medicine and Public Health, School of Medicine, University of Valencia, 46010 Valencia, Spain; jose.sorli@uv.es (J.V.S.);; 2 CIBER Fisiopatología de la Obesidad y Nutrición, Instituto de Salud Carlos III, 28029 Madrid, Spainoscar.coltell@uji.es (O.C.); 3 Servicio de Oftalmología, Hospital Clínico Universitario Lozano Blesa, 50009 Zaragoza, Spain; 4 Department of Medical Oncology, University Clinic Hospital of Valencia, 46010 Valencia, Spain; 5 Biomedical Research Networking Centre on Cancer (CIBERONC), Health Institute Carlos III, 28029 Madrid, Spain; 6 INCLIVA Biomedical Research Institute, 46010 Valencia, Spain; 7 Department of Physiology, School of Medicine, University Antonio Nariño, Bogotá 111511, Colombia; 8 Cardiovascular Risk and Nutrition Research Group, Hospital del Mar Medical Research Institute (IMIM), 08003 Barcelona, Spain; 9 Department of Computer Languages and Systems, Universitat Jaume I, 12071 Castellón, Spain

**Keywords:** nutrition, aging, mediterranean diet, chronotype, mental health, chronic diseases, depression, methylation clocks

## Abstract

Background and objectives: Depression often results in premature aging, which increases the risk of other chronic diseases, but very few studies have analyzed the association between epigenetic biomarkers of aging and depressive symptoms. Similarly, limited research has examined the joint effects of adherence to the Mediterranean diet (MedDiet) and chronotype on depressive symptoms, accounting for sex differences. Therefore, these are the objectives of our investigation in a Mediterranean population at high cardiovascular risk. Methods: We analyzed 465 older subjects (aged 55–75) with metabolic syndrome and assessed depressive symptoms by the Beck Depression Inventory (BDI-II). MedDiet adherence was measured with the 17-item MedDiet score, and chronotype with the Morningness–Eveningness Questionnaire (MEQ). Blood DNA methylation was analyzed, and epigenomic biomarkers of age acceleration were determined. We focused on the Dunedin Pace of Aging Computed from the Epigenome (DunedinPACE). We fitted multivariable models with interaction terms. Results: Prevalence of depression was statistically higher in women (*p* < 0.001). MedDiet adherence was strongly and inversely associated with depressive symptoms in the whole population (*p* < 0.01), while the MEQ score was inversely associated (*p* < 0.05). In the joint analysis, both MedDiet adherence and chronotype remained statistically associated with the BDI-II score (*p* < 0.05), showing additive effects. No interaction effects were observed. In women, a higher score in depressive symptoms was significantly associated with faster age acceleration (measured with the DunedinPACE biomarker). This association remained significant even after adjustment for MedDiet adherence and chronotype. Conclusions: In older subjects with metabolic syndrome, the eveningness chronotype was associated with greater depressive symptoms, but a higher adherence to the MedDiet could potentially counteract the chronotype risk with additive effects. Women showed stronger associations, and importantly, we reported for the first time in this population that depressive symptoms were associated with accelerated aging.

## 1. Introduction

The most prevalent risk factor for a wide range of chronic diseases is aging [1]. The aging of populations poses novel challenges to health, long-term care, and welfare systems. The promotion of healthy aging is of the utmost importance in light of the global trend of population aging [2,3,4]. To promote healthy aging, it is important to take into consideration the broad definition of health, which encompasses the best state of physical, mental, and social health [5,6]. Depression, a mood disorder affecting mental wellness, is a common mental health issue among elderly individuals and represents an increasing public health concern [7]. The prevalence of depression varies by country, but it is estimated that approximately 14% of the global elderly population aged 60 and over suffer from mental problems. Rong et al. published the estimated prevalence and trends of depressive disorders among older persons globally, regionally, and nationally across more than 150 countries during the past 30 years [7]. Moreover, for senior individuals, depression is often under-diagnosed in primary care settings [8,9]. Depression is linked to distress, which may result in limitations in physical, mental, and social health. It frequently adversely impacts the progression and complicates the management of other ongoing medical conditions [10]. On the other hand, the incidence of some chronic diseases such as cancer or cardiometabolic diseases can increase the risk of depression [11,12,13].

Consequently, further investigation is required into the characteristics and risk factors linked to depression in the older population, while also integrating recent methodological advancements. In recent years, omics technologies have advanced considerably, facilitating the collection of genomic and epigenomic data. Numerous research has been undertaken on the genetics of depression, including large genome-wide association studies (GWAS) [14,15]; nonetheless, the findings remain relatively inconsistent [16], potentially due to substantial interaction with environmental factors [17,18]. The application of epigenomics, particularly research on DNA methylation, appears more promising [19]. Among the epigenetic analyses, those investigating the so-called accelerated aging through the computation of the so-called “epigenomic clocks” are generating the most interest [20,21,22,23]. Research into the discovery of novel methylomic clocks is undergoing enormous growth and application to diverse health issues.

Currently, the most commonly used clocks are those known as first- (Horvath and Hannum clocks) [24] and second-generation (GrimAge and PhenoAge) clocks [25,26]. These methylomic clocks estimate a person’s biological age and also calculate the so-called accelerated aging as the difference between chronological age and biological age. Many of them have been associated with a higher risk of chronic diseases including diabetes, cardiovascular diseases, cancer and total mortality [24,25,26,27,28]. In addition to these clocks, the DunedinPACE (Dunedin Pace of Aging Computed from the Epigenome) is the most notable of the new generation [29]. This clock measures the pace of biological aging, and there are some investigations showing associations with cardiovascular diseases diabetes, cancer, mortality, and liver and kidney diseases [29,30,31]. Nonetheless, given that DunedinPACE is more recent, there is a paucity of studies utilizing it in comparison to its predecessors. It is intriguing that, despite the hypothesis that depression may be associated with greater biological aging, there are very few studies that have investigated this link. Furthermore, inconsistent findings have been observed in some investigations [32,33,34,35,36,37,38]. For this reason, we are interested in testing this association in our elderly population. There are several factors that can explain these differences between studies. Among them, we can mention the heterogeneity in the definition of depression [16], demographic characteristics [39,40], and other lifestyle factors [41,42,43]. Regarding the demographic characteristics, studies consistently show a higher prevalence of depression in women than in men [39,40]. Therefore, in the present investigation, we will conduct sex-stratified analyses.

Of the lifestyle factors, one of the most examined is diet [44,45,46,47,48,49,50,51]. In general, we can state that a healthy diet, particularly the Mediterranean diet (MedDiet), is associated with a lower risk of depression [44,47,50], but there are also some contradictory results [51]. Another factor that is generating great interest is chronotype. Chronotype is the term used to describe an individual’s circadian rhythm, which dictates when they are most wakeful and active, as well as when they are most likely to fall asleep [52]. On a continuum from “extreme morning types” to “extreme evening types”, individuals can be classified in five or three groups. Psychiatric disorders are believed to be more prevalent among individuals with evening chronotypes. Regarding depression, a large body of evidence links eveningness to depressive disorders [53,54,55,56,57,58,59], although some heterogeneity by sex [58] and population characteristics has been reported [59]. Thus, the present study aims to address these gaps in an older Mediterranean population involving chronotype, adherence to MedDiet, and accelerated aging. To accomplish this goal, first, we will test the independent and joint associations between chronotype and adherence to MedDiet with depression and depressive symptoms, considering the sex heterogeneity. Secondly, we will examine the extent to which depressive symptoms are associated with accelerate aging in this population, both before and after taking into account chronotype and MedDiet adherence.

## 2. Materials and Methods

### 2.1. Study Design and Participants

We conducted a cross-sectional analysis at baseline involving participants from the PREDIMED Plus-Valencia study (*n* = 465), a field center of the multi-center PREDIMED Plus study; an ongoing randomized primary prevention trial in Spain [60]. We collected data and samples during the baseline visit for each participant. We recruited participants consecutively from March 2014 to December 2016. No longitudinal follow-up was conducted in this study. The trial was registered at https://www.isrctn.com/ISRCTN89898870 (accessed on 1 December 2024). The eligible participants, recruited from several primary care health facilities in the Valencia field center, were community-dwelling adults (men, 55–75 years; women, 60–75 years) with body-mass index (BMI) in the overweight or obesity range and metabolic syndrome. The inclusion and exclusion criteria have been published elsewhere [60]. Briefly, the inclusion criteria included the above-mentioned age range, the presence of overweight or obesity (BMI for overweight: 27–29; for obesity: 30–39 kg/m^2^), and people who, at baseline, met at least three components of the metabolic syndrome and had no cardiovascular disease at enrollment. The exclusion criteria included the following: inability to give written informed consent or communicate with study staff; institutionalization; history of previous cardiovascular diseases; active malignant cancer; any severe co-morbid condition with less than 24-month life expectancy; impossibility to follow the recommended interventions; body weight loss > 5 kg during the 6 months prior to the screening visit; food allergy to any MedDiet component; immunodeficiency or HIV-positive status; liver cirrhosis or chronic renal failure; current use of weight loss medication; concurrent participation in another randomized clinical trial; and any other condition that may interfere with the completion of the study protocol.

We conducted the analysis on these participants, recruited in the Valencia field center, because the chronotype data and DNA methylation results [61] had been assessed only at the Valencia site. The demographic, clinical, lifestyle, and depression data were obtained from all participants recruited at the Valencia site (*n* = 465). From them, additional chronotype data (not included in the general multicenter study) were obtained (*n* = 426). DNA methylation analyses and the calculation of accelerated aging were performed for 414 subjects.

Participants provided written informed consent. The study protocols and procedures were approved according to the ethical standards of the Helsinki Declaration by the Human Research Ethics Committee of Valencia University, Valencia (approval codes H1373255532771, 15 July 2013; and H1509263926814, 6 November 2017).

### 2.2. Baseline Demographic, Clinical, Anthropometric, Biochemical, and Lifestyle Variables

At baseline, socio-demographic data, lifestyle (smoking, sleep duration, etc.), medication, and clinical characteristics were assessed by a questionnaire, as previously described [60]. The depression diagnosis was obtained through this questionnaire and was defined as a self-reported lifetime medical diagnosis of depression [45,46]. In addition, we gathered the age at which depression received the diagnosis. Type 2 diabetes was assessed as previously reported [60]. Anthropometric measurements and blood pressure were assessed by trained personnel following the PREDIMED Plus protocol [62,63]. Weight and height were assessed using calibrated scales and a wall-mounted stadiometer, respectively. The Body Mass Index (BMI) was determined by dividing weight in kilos by the square of height in meters. Waist circumference was measured at the midpoint between the lowest rib and the iliac crest, following normal exhalation, with an anthropometric tape. Blood pressure was determined using a validated semiautomatic oscillometer (Omron HEM-705CP, Netherlands) while the individual remained seated for 5 min. After an overnight fast, venous blood samples were collected by venipuncture. Fasting plasma total cholesterol, HDL-C, LDL-C, triglycerides, and glucose were determined as previously described [63]. A complete blood count (CBC) in peripheral blood samples (including the total leukocyte count and the types of white blood cells) was additionally determined as previously described [61].

The validated REGICOR short Physical Activity questionnaire [64] was employed to measure leisure-time physical activity. The questionnaire included questions regarding the type of activity, frequency (number of days), and duration. The total energy expenditure associated with leisure-time physical activity was calculated as the sum of the frequency, duration, and intensity of each activity, divided by 30 days, and was estimated in Metabolic Equivalent of Tasks (METs)/min/day as a continuous variable. A dichotomous variable for physical activity was also derived, which was based on the median values. We estimated adherence to the MedDiet using the validated 17-item screening questionnaire for the PREDIMED-Plus study [65]. This questionnaire consisted of 17 questions about compliance with dietary habits reflecting MedDiet. We scored the questions 0 (did not meet criteria) or 1 (achieved criteria), resulting in a maximum score of 17, with higher scores indicating greater adherence to the MedDiet. This scale is an updated version of the 14-item MedDiet adherence questionnaire [14-MEDAS], previously validated by our group [66]. Both scales share most items. The updated version [17-MEDAS] added a few additional items and an energy-reduced MedDiet framework. We have published the detailed questions that comprise the 17-MEDAS score [61]. Subsequently, the score was categorized into categorical variables: one based on the mean of the population (less than or higher than 8 points) and another categorized into three approximate tertiles: low (0–6) medium (7–9), and high adherence (10–17 points).

### 2.3. Depressive Symptoms’ Evaluation

We selected the Beck Depression Inventory (BDI-II) [67], which has been validated in Spain [68], to assess depressive symptoms at baseline. This is one of the most widely used and well-validated self-report measures of depressive symptoms in adolescents and adults (from 13 to 80 years), providing a current measurement of depression-related symptoms. The BDI-II consists of a 21-item questionnaire utilizing a Likert-type response scale for each item ranging from 0 (not present) to 3 (all of the time). The sum of all responses yields the total score, which spans from zero to 63. Higher total scores indicate more severe depressive symptomatology. There are some standardized cut-offs to classify individuals depending on the total score reached: minimal depression (0–13), mild depression (14–19), moderate depression (20–28), and severe depression (29–63) [69].

### 2.4. Morningness–Eveningness Questionnaire (MEQ)

We assessed individual variability in diurnal preference or chronotype using the widely used morningness–eveningness questionnaire [70]. The MEQ is considered an international gold standard measure of chronotype and is one of the most predominantly used measures in chronobiology. The MEQ comprises 19 items that pertain to preferred wake-up and bedtimes, as well as daily activity schedules. A higher total score indicated morningness. We used the MEQ score as a continuous variable for some analysis, taking into account the potential differences in the cut-off points reported in different studies [71]. The originally proposed MEQ cut-offs for chronotypes were as follows: definitely morning type (70–86 points), moderately morning type (59–69 points), intermediate type (42–58 points), moderately evening type (31–41 points), and definitely evening type (19–30 points). We also grouped extreme and moderate morning and evening types for further analysis (less than 42 were evening types and more than 58 were morning types).

### 2.5. DNA Methylation Analysis and DunedinPACE Calculations

Genomic DNA was isolated from blood. We measured the quantity of double-stranded DNA using PicoGreen (Invitrogen Corporation, Carlsbad, CA, USA). As previously reported [61], we analyzed DNA methylation using the Infinium HumanMethylationEPIC BeadChip (850 K) array (Illumina Inc., San Diego, CA, USA). We additionally processed the arrays at the Human Genomics Facility at Erasmus MC in Rotterdam, the Netherlands, which included bisulfite conversion using the Zymo EZ-96 DNA Methylation Kit (Zymo Research, Irvine, CA, USA), hybridization, and scanning using an Illumina HiScan machine. We also conducted quality control procedures to assess the quality and reliability of the DNA methylation data obtained using the R packages Minfi (release 3.18), Meffil (release 1.1.1), and ewastools (release 1.7.2) [72,73,74]. We successfully performed quality control on a total of 414 samples, after which we computed DNA methylation clocks. We carried out data normalization [75,76], including functional normalization and normal-exponential out-of-band (NOOB) correction, and obtained beta values for the corresponding CpG sites. DunedinPACE scores were calculated from the beta matrices using the DunedinPACE R package as described by Belsky et al. [29]. Although our main aim was to analyze the depression associations with accelerated aging using the DunedinPACE biomarker, considering that it is a novel biomarker and that previous studies analyzed second-generation biomarkers such as the GrimAge epigenetic clock and age acceleration (AgeAccelGrim) [25], we also computed this biomarker from the beta values according to the algorithm proposed by Lu et al. [25] using the online Horvath epigenetic age calculator [24].

### 2.6. Statistical Analysis

Before testing the study’s hypotheses, we completed the initial screening and cleaning of the data. We implemented chi-square tests to analyze proportions. Students’ *t*-tests and ANOVA tests were applied to compare crude means of continuous variables. We analyzed the associations between the factor of interest and the dependent variables (DBI-II-score, morningness–eveningness, or age acceleration) using multivariable regression models sequentially adjusted for potential confounders. When indicated, models were sequentially adjusted as follows: model 1, unadjusted; model 2, adjusted for sex, age, and diabetes; model 3, additionally adjusted for BMI, smoking, physical activity, sleep duration, and education. Furthermore, depending on the statistical analysis, models were additionally adjusted for depression diagnosis, for chronotype, and/or for adherence to the MedDiet. For the study of interactions between the chronotype and adherence to the MedDiet we used multivariable hierarchical regression models including the main effects and the interaction terms and additionally adjusted for the above-mentioned covariates. For accelerated aging, we focused our analysis on the Dunedin-PACE score as the dependent variable, and we fitted models sequentially adjusted for the potential confounders as indicated. Additionally, and only for comparative purposes with other studies, we analyzed the AgeAccelGrim biomarker as the dependent variable. We used general linear models for continuous variables as the dependent variable. We used multivariable logistic regression models for dichotomous variables as outcomes. We estimated the adjusted means for the continuous variables from the corresponding multivariate corrected models. We conducted analyses for the entire population, stratifying them based on sex. We performed statistical analyses using IBM SPSS Statistics version 26.0, NY, and specific tools in R. All tests were two-tailed, and *p*-values < 0.05 were considered statistically significant for these associations.

## 3. Results

### 3.1. General Characteristics of Participants and Prevalence of Depressive Symptoms

At baseline, Table 1 displays the demographic, lifestyle, and clinical characteristics of the study participants (*n* = 465) by sex. This represented the whole sample size of participants recruited in the PREDIMED-Plus-Valencia study, which included both men (*n* = 198) and women (*n* = 267). They all had metabolic syndrome, and the mean age was 65 years (SD: 5 years). The prevalence of obesity was high, and there were no statistically significant differences between men and women (*p* = 0.651 for BMI). Similarly, we did not observe any statistically significant differences in the prevalence of diabetes based on sex (*p* = 0.753). We also did not find statistically significant differences in adherence to the MedDiet by sex (*p* = 0.022). However, we did find statistically significant differences between men and women (*p* < 0.05) in the level of education (predominantly up to primary education for women), in tobacco smoking (less consumption among women), and in physical activity (more sedentary among women). Regarding the prevalence of previously diagnosed depression, it was 8% of the total population, and the average DBI-score was 9 points. (SE: 0.2). While 77% of participants reported “minimal depressive symptoms”, 15.5% of the participants reported “mild depressive symptoms”, 5.2% “moderate”, and 1.5%, “severe depressive symptoms”.

For all these variables of depression and the level of depressive symptomatology, we have found very significant differences by sex, with values always higher in women (*p* < 0.05). However, for diagnosed depression, we additionally gathered the age of onset for any lifetime depressive disorder. In this population, women were not likely to have an earlier onset of depression compared to males, as no statistically significant differences were observed in the mean age at onset of depression between men (51.8; SE: 2.5 years) and women (50.3; SE: 2.5 years); *p* = 0.751. When we analyzed sleep duration, it was shorter in women than in men (*p* < 0.05). Similarly, the average score of their MEQ questionnaire was also lower (*p* < 0.05), indicating a more evening chronotype. Chronotype data were collected from only 426 participants who provided complete responses to all items on the MEQ, which was administered only at the Valencia site.

### 3.2. Association of Depressive Symptoms with Demographic, Clinical and Lifestyle Variables

First, we tested the internal consistency of the BDI-II scale in this population and obtained an excellent result. We found that our participants had a Cronbach’s alpha of alpha = 0.87. Second, we analyzed the association between the main demographic, clinical, and lifestyle characteristics and depressive symptoms in both men and women. Table 2 presents the results of a multivariable regression model, adjusted for the other variables.

Sex was strongly associated with the severity of depressive symptoms (*p* < 0.001). Women had an average regression coefficient 3.23 (SE: 0.68) points higher than men, even after the adjustment of the other covariates. Similarly, having a previous medical diagnosis of depression was associated with higher levels of depressive symptoms (beta: 3.12; SE: 1.04; *p* = 0.003) in the multivariable model taking into account the adjustment for sex. Type 2 diabetes was also significantly associated with higher levels of depressive symptoms (1.64: SE: 0.06 points in diabetic subjects compared to non-diabetics; *p* = 0.005). We did not find a statistically significant association between education, smoking, physical activity, BMI, and age and depressive symptoms after the multivariable adjustment, including sex. Interestingly, sleep duration was inversely associated with depressive symptoms (*p* = 0.010). Although we measured sleep duration both on the workdays and on the free days, due to the high correlation of these variables and to prevent multicollinearity, we only included one of them (sleep duration on the workdays) in the fitted model. Further, we estimated the association of the above-analyzed variables with diagnosed depression in a multivariable logistic regression model including all of them. In this model, only sex was significantly associated with lifetime clinical diagnosis of depression (OR = 3.11; 95% CI: 1.28–7.61; *p* = 0.01 for women versus men).

### 3.3. Association of Chronotype with Depression and Depressive Symptoms

**Figure 1:**
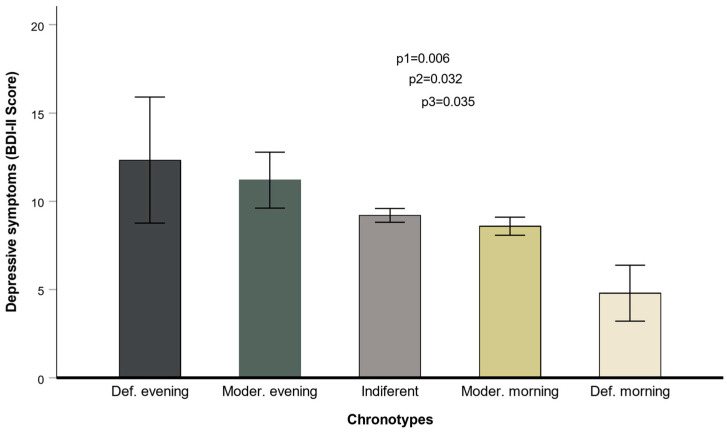
Association between the five chronotypes and depressive symptoms in men and women (*n* = 426). *p*-values for the chronotype variable are shown depending on the covariates. 1: unadjusted; 2: model adjusted for sex, age, and diabetes; 3: additionally adjusted for BMI, smoking, physical activity, sleep duration, and education. Error bars: SE of means.

The total scores of the MEQ in this sample ranged from 26 to 76 points, with higher values indicating a morningness chronotype. The internal consistency of the MEQ in the whole sample was good (Cronbach’s alpha = 0.727). We derived the five categories for the chronotypes considering the MEQ-score cut-offs indicated in the Methods. Thus, in the whole sample, the prevalence of the five categories for chronotypes were as follows: definitely morning type (3.5%), moderately morning type (33.3%), intermediate type (58.9%), moderately evening type (3.6%), and definitely evening type (0.7%). We detected a strong association between the morningness–eveningness scores and the depressive symptoms according to the BDI inventory (Figure 1). Mean BDI scores for each chronotype were 4.8 ± 0.8; 8.6 ± 0.8; 9.2 ± 0.4; 11.2 ± 2.4, and 12.3 ± 4.9 points for the definitely morning, moderately morning, intermediate, moderately evening, and definitely evening types, respectively (*p*-trend = 0.005). This association remained statistically significant after adjustment for sex, age, and diabetes (*p* = 0.032) and even after additional adjustment for BMI, smoking, physical activity, sleep duration, and education (*p* = 0.035).

**Figure 2:**
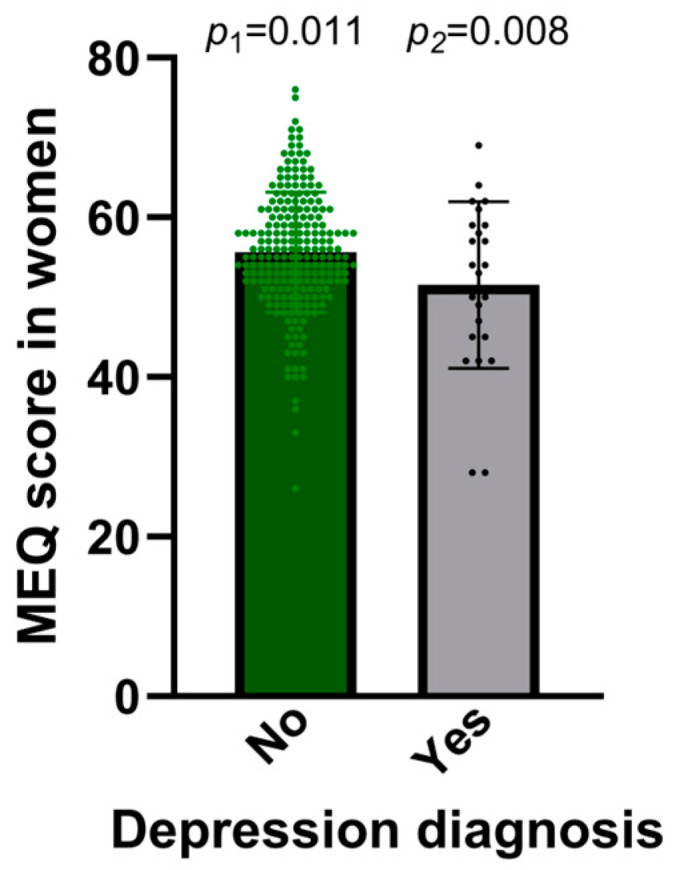
Association between depression diagnosis (No, Yes) and the Morningness–Eveningness Questionnaire (MEQ) score in women (*n* = 247). *p*-values for depression in models 1 (adjusted for age and diabetes) and 2 (additionally adjusted for BMI, smoking, physical activity, sleep duration, and education. Means and dots are depicted. Error bars: SE of means.

We also grouped extreme and moderate morning and evening types for further analysis and created a three-category variable (evening type, intermediate type, and morning type). This variable was also strongly associated with levels of depressive symptoms. Thus, the prevalence of severe depression was 11.1%, 1.6%, and 0.0% in subjects with the evening, intermediate, and morning chronotypes, respectively, whereas the prevalence of minimal depression was 61.1%, 77.7%, and 81.5% in subjects with the evening, intermediate, and morning chronotypes, respectively (*p* < 0.047). The stratified analysis by sex for current depressive symptoms indicated similar trends in both men and women. Additionally, we examined an association between a lifetime diagnosis of depression and the MEQ-score examined as a continuous variable. Initially, we tested the association in the entire population, including both men and women. In the unadjusted model, the MEQ score exhibited a significant association (beta = −2.92 ± 1.40 points; *p* = 0.023) among patients with diagnosed depression compared to those without a diagnosis. Nonetheless, following adjustments for sex, age, and diabetes, the statistical significance was diminished (*p* = 0.052). The stratified model by sex revealed a stronger and statistically significant association in women (*p* = 0.011) compared to men (*p* = 0.551). Figure 2 shows the association between the MEQ score (as a continuous variable) and previously diagnosed depression in women. This model (adjusted for age and diabetes) indicates that women with a prior diagnosis of depression scored −4.25 ± 1.70 points lower on the MEQ compared to those without such a diagnosis. This association remained statistically significant following further adjustments for BMI, smoking, physical activity, sleep duration, and education (*p* = 0.008).

### 3.4. Association of Adherence to MedDiet with Depressive Symptoms

**Figure 3:**
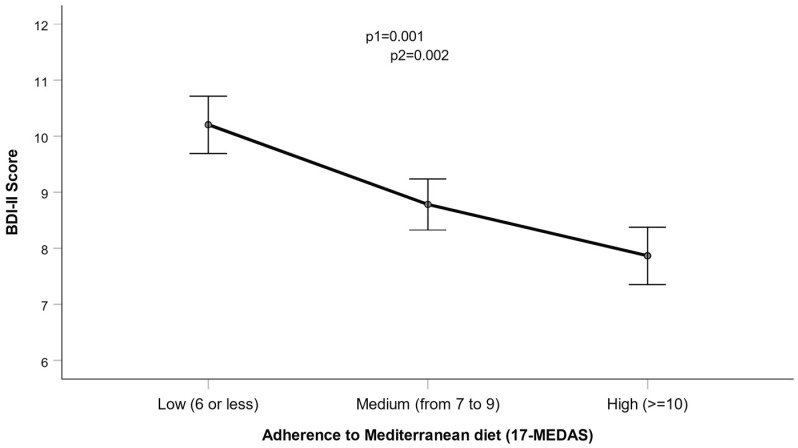
Association between adherence to the Mediterranean diet (three categories) and depressive symptoms (BDI-II score) in men and women (*n* = 465). 1: model adjusted for sex, age, and diabetes. 2: model additionally adjusted for BMI, smoking, physical activity, sleep duration, and education. Error bars: SE of means.

First, we analyzed the association between adherence to the MedDiet as a continuous variable and depressive symptoms in both men and women together. We used the 17-MEDAS score, where a higher score indicates greater adherence. In all the models used, adherence to the MedDiet had a highly significant inverse association with depressive symptoms (beta = −0.34 ± 0.10 points decrease in depressive symptoms per 1-point increase in adherence; *p* = 0.001 in a model adjusted for sex, age, and diabetes). This result remained statistically significant even after additional adjustment for BMI, smoking, physical activity, sleep duration, and education (beta = 0.35 ± 0.10: *p* = 0.001). In the sex-stratified analysis, the findings of the inverse association were comparable in both men and women but more pronounced in women. Figure 3 illustrates the association between the TDI-II score and adherence to the MedDiet, categorized into three levels: low, medium, and high, as detailed in the methods section. Once again, our model adjusted for sex, age, diabetes, BMI, smoking, physical activity, sleep duration, and education revealed a strong inverse association with depressive symptoms (*p* = 0.002).

When we looked at the association between adherence to MedDiet (as continuous) and having been diagnosed with depression at some point in the life, we did not find a statistically significant *p*-value for the whole population (*p* = 0.142 for the fully adjusted model). Likewise, we did not find a statistically significant association with depression diagnosis in either men (*p* = 0.531) or women (*p* = 0.056).

### 3.5. Joint Effect of the Chronotype and of the Adherence to MedDiet in Depressive Symptoms

After examining the separate effect of chronotype and adherence to MedDiet on depressive symptoms, now we have analyzed the joint effects including both variables in multivariate regression models. Table 3 presents the results of the statistical models analyzing these joint effects (beta ± SE and *p*-values). Models 1, 2, and 3 have the same sequential fit as those used for the separate effects.

The chronotype and adherence to the MedDiet variables both remained statistically significant in all adjusted models, each being an independent predictor of depressive symptomatology. In the fully adjusted model (model 3), both variables presented statistically significant additive effect, such that a morning chronotype was associated with lower depressive symptoms (−0.89 ± 0.45; *p* = 0.046) for each category of chronotype advancement towards the morning. At the same time, greater adherence to the MedDiet would also contribute to a decrease in depressive symptoms (−0.29 ± 0.11 points; *p* = 0.007) for each point increase in adherence. After comparing the main effects, another regression model was additionally adjusted including the interaction terms between chronotype and MedDiet. Nonetheless, we did not see statistically significant interactions, suggesting an absence of heterogeneity in the additive joint effects in the three models fitted.

### 3.6. Association Between Depressive Symptoms and Age Acceleration

**Figure 4:**
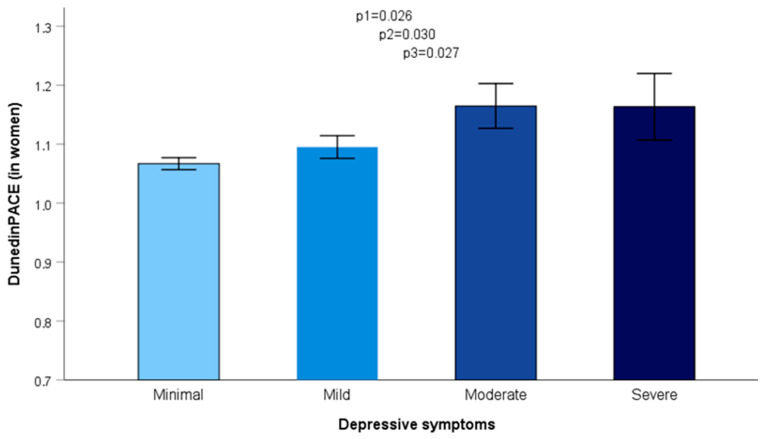
Association between depressive symptoms (4 standardized categories) and age acceleration (DunedinPACE biomarker) in women (*n* = 216). 1: model adjusted for sex, age, and diabetes. 2: model additionally adjusted for BMI, smoking, physical activity, sleep duration, and education. 3: model additionally adjusted for chronotype and Mediterranean diet. Error bars: SE of means.

As indicated in the Methods, we calculated the DunedinPACE scores as our selected biomarker for age acceleration. It is a novel blood DNA methylation biomarker that measures the rate of biological aging from a single blood sample and indicates the rate at which an individual ages in comparison to the standard rate of one year of aging for each calendar year. Values above 1 indicate “faster” biological aging, whereas values below 1 indicate “slower” biological aging. DNA methylation data and the subsequent DunedinPACE biomarker estimations were available for 414 participants. In this sample, DunedinPACE scores ranged from 0.66 to 1.53. First, we analyzed the association between depressive symptoms (BDI-II score) and the DunedinPACE biomarker in both men and women. However, after adjusting for sex, age, and diabetes, we obtained results that were borderline significant (*p* = 0.078). Then, we did a separate analysis for men and women and found a statistically significant association between higher BDI-II scores and faster aging in women (beta = 0.003 ± 0.001; *p* = 0.040). No statistically significant results were obtained in men (*p* = 0.905) in the same regression model adjusted for sex, age, and diabetes. Further, we tested the association between the severity of depressive symptoms and the DunedinPACE biomarker for age acceleration in both men and women. In women (*p* = 0.026), but not in men (*p* = 0.289), we obtained a statistically significant association in the model adjusted for age and diabetes (Figure 4). This association remained statistically significant after additional adjustment for BMI, smoking, physical activity, sleep duration, and education.

Moreover, when additional adjustments for the chronotype and adherence to the MedDiet were considered, the association between higher severity of depressive symptoms and accelerated aging (DunedinPACE biomarker) remained statistically significant (*p* = 0.027). Finally, with comparative purposes, we analyzed the association between depressive symptoms and age acceleration using the classic GrimAge epigenetic clock and age acceleration (AgeAccelGrim) as described in the Methods. For this biomarker, we observed a similar trend consisting of an increase in age acceleration associated with the severity of depressive symptoms, but results did not reach statistical significance (*p* = 0.375 in the whole population and *p* = 0.132 in women).

## 4. Discussion

This study, conducted among older persons at high cardiovascular risk within a Spanish Mediterranean population, demonstrated the importance of chronotype in terms of depressive symptoms, in agreement with other investigations [59,77]. We observed an increased presence of depressive symptoms associated with a later chronotype [52,53,54,55,59,77]. Chronotype is believed to influence mental health [78,79], with current evidence indicating that persons with evening chronotypes exhibit an increased risk of mental health disorders, mainly depressive symptoms [52,53,54,55,59,77,78,79,80,81,82]. Nevertheless, the underlying mechanisms remain under investigation [83,84,85], and further research is required. We investigated both the separate and the joint effects of MedDiet and chronotype on depressive symptoms in this population and identified a robust association. Higher adherence to MedDiet was strongly and inversely associated with depressive symptomatology, whether the MedDiet was analyzed as a continuous or categorical variable. Numerous prior studies have examined the association between diet and depressive symptomatology [43,44,45,46,47,48,49,50,51]. The results are mixed depending on the diet [48,49,50,51], and many factors have been identified as potential confounders. A contributing factor may be the heterogeneous definitions of depression or depressive symptoms [16], together with the variability in the definitions of the dietary patterns or components examined [48,49]. Depression and depressive symptomatology have been reported to be a “minimally phenotypic” condition in many epidemiological studies related to nutrition or to genetics, relying on brief questionnaires or self-reported classifications [16]. In addition, cut-off points for depression definition based on the BDI-II score may be different depending on the published study [16,68,86]. Thus, for example, even in prior nutritional studies conducted out in the PREDIMED-Plus cohort, multiple BDI-II cut-off values to identify depression have been considered [45,46,69]. Although the heterogeneity in the definition of depression and depressive symptomatology also affects studies that analyze chronotype and all the factors that are going to be related to depression, the results of the association between being an evening type with more severe depressive symptoms are more robust than the association of diet with depressive symptoms. The fact that the chronotype is a more stable measure across time may contribute to this. According to the results of a longitudinal study examining the stability of chronotype over 7 years [87], the authors found that chronotype was a stable, trait-like construct with only a minor level advance over the analyzed period. Although it is known that, as people age, they go towards a more morning chronotype [88], in general, people have more stable chronotypes than diets since the chronotypes are more difficult to modify. However, diet is a more dynamic factor; its composition can shift day to day. Furthermore, it is challenging to determine whether diet is the cause or the consequence in the context of depression. The difference between dietary flexibility and chronotype stability might clarify certain findings in this study. Thus, we found statistically significant associations between chronotype and the variable of diagnosis of depression. This is a self-reported medical diagnosis over a lifetime (ranging from 20 to 70 years in this sample). However, the association between MedDiet and diagnosed depression diminished in statistical significance in comparison with its strong association of MedDiet with current depressive symptoms. There are additional factors that contribute to the comprehensive definition of a dietary pattern or dietary characteristics, in addition to the stability of the chronotype and the variability of the diet. The validity in the dietary definition is an important aspect in the results of previous meta-analysis that studied the relationship between diet and depressive symptoms [48,49,89,90,91]. Consequently, diets that are vaguely defined, such as those that are based on macronutrients, dietary diversity, or a Western pattern, have an inconsistent association with depression phenotypes. Conversely, traditional diets, including MedDiet and other healthy dietary patterns, exhibit more robust inverse associations [48,49,50,89,90,91]. In the present study, we have observed a strong inverse association between depressive symptomatology and adherence to the MedDiet using the 17-MEDAS [65]. In this Mediterranean population, this questionnaire has been validated explicitly [65] and is an updated version of the 14-MEDAS, which was previously validated by our group [66]. This updated version provides additional evidence to support the statistical associations.

However, this is a cross-sectional study, and additional longitudinal studies analyzing depression incidence are needed [92]. Moreover, although the results from intervention trials with MedDiet support a protective role of this healthy dietary pattern [93], more studies stratified by sex, age group, geographical origin, and other conditions will provide additional evidence for more personalized interventions. One relevant finding of our cross-sectional study is that we have simultaneously examined the effects of adherence to MedDiet and the chronotypes using MEQ. This is the sole site in the PREDIMED-Plus multicenter study that has the chronotype measured at baseline. In the multivariable regression model, which includes other potential confounders, we have observed additive and independent effects for both the chronotype and the MedDiet. Thus, higher adherence to the MedDiet could counteract the adverse association of the evening chronotype with higher risk of depression.

Among the other factors highly associated with depression and depressive symptoms in the present study, we would like to highlight sex. Women have a more than two-times-higher prevalence of diagnosed depression and significantly higher severity of depressive symptoms than men. This sex-specific association has been reported in several previous studies [39,40,94]. More research is still needed to better explain such complex sex-specific differences, perhaps related to the fact that women are more likely to have internalizing disorders such as depression and men more likely to have externalizing disorders such as antisocial personality. We also have observed some sex-specific differences in the investigated associations (higher in women) in this study, mainly related to the chronotype and biological age acceleration.

To our knowledge, this is the first study analyzing the association between depressive symptoms and age acceleration in a Mediterranean population in Spain. Likewise, at the global level, there are still very few studies analyzing the association between epigenetic age acceleration/pace of biological aging and depressive symptoms [32,33,34,35,36,37,38]. Currently, there is a great interest in the biomarkers of biological aging and in the estimation of age acceleration, considering that several studies have demonstrated a strong association between the epigenetic biomarkers of aging for age acceleration and higher risk of chronic diseases and mortality [20,21,22,23,24,25,26,27,28,29,30,31]. Moreover, epigenetic biomarkers based on DNA methylation are dynamic and can be modified by specific interventions [95]. However, the studies undertaken have yielded mixed results. Again, the heterogeneity of the depression definition, the socio-demographic characteristics of the population, the adjustment for potential confounders, and the diversity of epigenetic clocks may have an influence on the inconsistent results. Thus, one of the first studies carried out on epigenetic biomarkers analyzed the three so-called first-generation clocks [32] in White and African American participants in the “Healthy Aging in Neighborhoods of Diversity across the Life Span study”. The three epigenetic biomarkers used were universal epigenetic age acceleration (AgeAccel), intrinsic epigenetic age acceleration, and extrinsic epigenetic age acceleration. However, no statistically significant associations were found between these biomarkers and depressive symptoms (BDI-II score). However, Liu et al. [33] analyzed the association between 6 DNA methylation biomarkers (including Horvath’s DNAm age acceleration, intrinsic epigenetic age acceleration Hannum’s DNAm age acceleration, extrinsic epigenetic age acceleration, GrimAge acceleration, and PhenoAge acceleration) and depressive symptoms in a co-twin control study accounting for the potential confounders (genetics and environmental) and obtained statistically significant association between some of the biomarkers (Hannum, extrinsic epigenetic age acceleration, and PhenoAge. Similar mixed results have been reported in other studies using the first- and second-generation DNA methylation biomarkers. However, the DunedinPACE biomarker [29], a third-generation biomarker, has been reported to outperform the previously mentioned biomarkers, and recent studies have obtained statistically significant associations with depression phenotypes using the DunedinPACE in comparison with the first- and second-generation biomarkers. So, Perret et al. [35] came up with five epigenetic biomarkers: Horvath’s pan-tissue (Horvath1), Horvath’s skin-and-blood (Horvath2), pediatric buccal-epigenetic age (PedBE), DunedinPACE, and stress response reactivity. However, only DunedinPACE showed significant associations with depression phenotypes. Taking into account the previous results, in our study, we focused our aims on the DunedinPACE biomarkers. Additionally, we estimated the GrimAge Acceleration biomarker for comparison purposes. As expected, we observed a strong association between the DunedinPACE biomarkers and depressive symptoms in women. A higher level of depressive symptoms was associated with faster age acceleration. This result was statistically significant in the model adjusted for age and diabetes and remained statistically significant after additional adjustment for BMI, smoking, physical activity, sleep duration, and education. The association was still statistically significant even after considering chronotype and MedDiet adherence, showing that depressive symptoms have their own effect on the DunedinPACE biomarker of accelerated aging. However, we did not obtain significant results when the same models were fitted using the GrimAge Acceleration biomarker despite obtaining a similar trend. Our results support previous research that first/second generation epigenetic clocks are not good predictors of depression or functional decline. They also support the use of the DunedinPACE biomarker as a beneficial way to find depression-related phenotypes, showing how important it is to choose the right epigenetic biomarker for aging research.

Our study has strengths and limitations. The main strengths are related to the well-phenotyped population, in terms of lifestyle variables, including the adherence to the MedDiet, the use of a validated and large version of the MEQ questionnaire, and the DBI-II questionnaire, as well as other covariates related to smoking, physical activity, sleep duration, and disease phenotypes. Moreover, we obtained DNA from blood and measured DNA methylation in an epigenomic-wide validated array and computed the most relevant biomarkers of aging, including the Dunedin-PACE and the GrimAge Acceleration biomarker. Despite these strengths, the study has some limitations. First, we have carried out a cross-sectional study, making it difficult to establish a causal relationship in the examined associations. However, we are collecting longitudinal data to better understand these and other longitudinal associations in the future. Second, additional studies should test the external validity of results in this older Mediterranean population at high cardiovascular risk. Future studies with large sample sizes should focus on the sex-specific associations suggested in our work to better understand the potential significance and mechanisms.

## Figures and Tables

**Table 1:** Demographic, clinical, and lifestyle characteristics of the study population according to sex.

	Total (*n* = 465)	Men (*n* = 198)	Women (*n* = 267)	*p*
Age (years)	65.2 ± 0.2	64.0 ± 0.4	66.2 ± 0.3	<0.001
Weight (Kg)	84.0 ± 0.6	92.4 ± 0.9	77.8 ± 0.6	<0.001
Waist (cm)	105.7 ± 0.5	110.9 ± 0.6	101.8 ± 0.6	<0.001
Body mass index (Kg/m^2^)	32.3 ± 0.2	32.2 ± 0.2	32.4 ± 0.2	0.651
Systolic blood pressure (mm Hg)	141.8 ± 0.8	144.2 ± 1.3	139.9 ± 1.1	0.012
Diastolic blood pressure (mm Hg)	80.9 ± 0.5	82.7 ± 0.7	79.6 ± 0.6	0.001
Total cholesterol (mg/dL)	196.6 ± 1.8	188.3 ± 2.8	202.8 ± 2.2	<0.001
HDL-C (mg/dL)	51.7 ± 0.5	47.2 ± 0.8	55.0 ± 0.7	<0.001
LDL-C (mg/dL)	124.7 ± 1.4	121.6 ± 2.3	127.0 ± 1.8	0.064
Triglycerides (mg/dL) ^1^	141.7 ± 2.8	139.4 ± 4.0	143.5 ± 4.0	0.475
Fasting glucose (mg/dL)	113.0 ± 1.3	113.8 ± 2.0	112.4 ± 1.6	0.584
Physical activity (MET-min/wk) ^2^	1676 ± 71	1919 ± 125	1497 ± 79	0.003
Sleep duration on workdays (h)	6.8 ± 0.1	7.0 ± 0.1	6.6 ± 0.1	0.001
Sleep duration on free days (h)	7.1 ± 0.1	7.3 ± 0.1	7.0 ± 0.1	0.001
Adherence to MedDiet (17-I) ^3^	8.0 ± 0.1	7.8 ± 0.2	8.2 ± 0.2	0.222
Chronotype (MEQ score) ^4^	56.0 ± 0.4	57.0 ± 0.6	55.2 ± 0.5	0.022
BDI-II score ^5^	9.0 ± 0.3	7.1 ± 0.4	10.4 ± 0.4	<0.001
Type 2 diabetes, n (%)	177 (38.1)	77 (38.9)	100 (37.5)	0.753
Depression diagnosis, n (%)	37 (8.0)	9 (4.5)	28 (10.5)	0.019
Educational level				<0.001
Primary, n (%)	288 (61.9)	97 (49.0)	191 (71.5)	
Secondary, n (%)	95 (20.4)	57 (28.8)	38 (14.2)	
University, n (%)	82 (17.6)	44 (22.2)	38 (14.2)	
Smoking				<0.001
Never smokers, n (%)	225 (48.4)	35 (17.7)	190 (71.2)	
Former smokers, n (%)	191 (41.1)	133 (67.2)	58 (21.7)	
Current smokers, n (%)	49 (10.5)	30 (15.2)	19 (7.1)	
Depressive symptoms ^6^				<0.001
Minimal (0–13)	362 (77.8)	177 (89.4)	185 (69.3)	
Mild (14–19)	72 (15.5)	16 (8.1)	56 (21.0)	
Moderate (20–28)	24 (5.2)	4 (2.0)	20 (7.5)	
Severe (29–30)	7 (1.5)	1 (0.5)	6 (2.2)	

Values are mean ± SE for continuous variables, n and (%) for categorical variables. *p*-value for the comparisons (means or %) between men and women. ^1^: ln-transformed for statistical testing. ^2^: Metabolic Equivalent of Task. ^3^: 17-item (17-I) questionnaire for adherence to Mediterranean diet (MedDiet). ^4^: Morningness–Eveningness Questionnaire; *n* = 426. ^5^: Beck Depression Inventory II. ^6^: Standardized cut-offs for depressive symptoms.

**Table 2:** Association between depressive symptoms and demographic, clinical, and lifestyle variables.

Parameter	Beta ± SE	*p*
*Sex*		
Women	(Ref.)	
Men	−3.23 ± 0.68	<0.001
Age (years)	0.02 ± 0.06	0.767
Body Mass Index (Kg/m^2^)	0.09 ± 0.06	0.289
*Type 2 diabetes*		
Yes	(Ref.)	
No	−1.64 ± 0.58	0.005
*Depression diagnosis*		
Yes	(Ref.)	
No	−3.12 ± 1.04	0.003
*Educational level*		0.184
University	(Ref.)	
Primary	1.15 ± 0.81	0.158
Secondary	−0.03 ± 0.92	0.972
*Smoking*		0.269
Current smokers	(Ref.)	
Former smokers	−0.89 ± 1.02	0.383
Never smokers	0.28 ± 0.99	0.779
Sleep duration on workdays (h)	−0.68 ± 0.26	0.010
*Physical Activity (Median)*		
High	(Ref.)	
Low	0.37 ± 0.59	0.533

Categorical variables are expressed in italics. Values are Beta ± SE (*n* = 465). Beta for each variable is the regression coefficient in the multivariable regression model for depressive symptoms, taking into account the joint effects of the other variables. *p* is the statistical significance of the corresponding beta.

**Table 3:** Joint effects of the chronotype and adherence to Mediterranean diet (MedDiet) in a multivariable regression model without interaction terms. Additional modelling for the interaction terms.

	Chronotype (C) ^1^ Main Effects		MedDiet (D) ^2^ Main Effects		Additional Model ^3^ with Interaction Terms (CxD)
Model	Beta (SE)	*p*	Beta (SE)	*p*	*p*_interaction_
Model 1 ^4^	−1.27 (0.46)	0.006	−0.29 (0.11)	0.009	0.458
Model 2 ^5^	−0.95 (0.50)	0.034	−0.29 (0.10)	0.006	0.502
Model 3 ^6^	−0.89 (0.45)	0.046	−0.29 (0.11)	0.007	0.352

^1^: Chronotype expressed as five levels (trend estimations for morningness) in a model also including the MedDiet adherence. Regression coefficients (beta) adjusted for the covariates indicated in each model. ^2^: Assessed by the 17-MEDAS as continuous in the model also including the chronotype. ^3^: After fitting the main terms, a new model, including the corresponding interaction terms between chronotype (C) and MedDiet (D), was tested. *p*-values for the interaction terms (CxD) depending on the covariates are shown. ^4^: Unadjusted. ^5^: Adjusted for sex, age, and diabetes. ^6^: Additionally adjusted for BMI, smoking, physical activity, sleep duration, and education.

## Data Availability

Neither the participants’ consent forms nor ethics approval included permission for open access. However, we follow a controlled data-sharing collaboration model, and data for collaborations will be available upon request pending application and approval. Investigators who are interested in this study can contact the corresponding author.
